# Different Waterlogging Depths Affect Spatial Distribution of Fine Root Growth for *Pinus thunbergii* Seedlings

**DOI:** 10.3389/fpls.2021.614764

**Published:** 2021-03-10

**Authors:** Saki Fujita, Kyotaro Noguchi, Takeshi Tange

**Affiliations:** ^1^Laboratory of Silviculture, Graduate School of Agricultural and Life Sciences, The University of Tokyo, Tokyo, Japan; ^2^Tohoku Research Center, Forestry and Forest Products Research Institute, Morioka, Japan

**Keywords:** fine root growth distribution, fine root morphology, fine root color, in-growth core, coastal forest restoration

## Abstract

The increase of waterlogged environments at forests and urban greenery is of recent concern with the progress of climate change. Under waterlogging, plant roots are exposed to hypoxic conditions, which strongly affect root growth and function. However, its impact is dependent on various factors, such as waterlogging depth. Therefore, our objective is to elucidate effects of different waterlogging depths on *Pinus thunbergii* Parl., which is widely used for afforestation, especially at coastal forests. We conducted an experiment to examine growth and morphology of fine roots and transpiration using 2-year-old seedlings under three treatments, (1) control (no waterlogging), (2) partial waterlogging (partial-WL, waterlogging depth = 15 cm from the bottom), and (3) full waterlogging (full-WL, waterlogging depth = from the bottom to the soil surface, 26 cm). As a result, fine root growth and transpiration were both significantly decreased at full-WL. However, for partial-WL, fine root growth was significantly increased compared to control and full-WL at the top soil, where it was not waterlogged. Additionally, transpiration which had decreased after 4 weeks of waterlogging showed no significant difference compared to control after 8 weeks of waterlogging. This recovery is to be attributed to the increase in fine root growth at non-waterlogged top soil, which compensated for the damaged roots at the waterlogged bottom soil. In conclusion, this study suggests that *P. thunbergii* is sensitive to waterlogging; however, it can adapt to waterlogging by plastically changing the distribution of fine root growth.

## Introduction

With the progress of climate change, it is predicted that precipitation regimes will change and extreme rainfall events will occur more frequently, consequently increasing waterlogged environments (Kundzewicz et al., 2014). As the diffusion of oxygen in water is 10,000 times slower than it is in air (Greenwood, 1961), oxygen availability for root respiration becomes limited under waterlogging (Blom and Voesenek, 1996), strongly affecting root growth and function. The risk of waterlogging stress is expected to increase at not only forests but also urban afforestation sites, as waterlogging occurs due to not only climate but also topography and soil properties, such as flat areas, clay soils, and poor drainage (Valipour, 2014).

Recently, coastal *Pinus thunbergii* Parl. forests have been gaining attention after the disastrous tsunami which occurred at pacific coast line of northeastern Japan in March 2011 (Sakamoto, 2012; Tanaka et al., 2013). Restoration of these sites is currently undergoing; however, in some cases, soil layers of the plantation growth base are of poor permeability and drainage due to the use of machinery (Ono et al., 2016), which results in waterlogged conditions after rainfall (Shinomiya et al., 2016). Fujita et al. (2020) reported that under waterlogging, fine root growth of *P. thunbergii* is severely inhibited. Furthermore, root damage such as partial root death and decay was observed from the decrease in root tissue density and darkening in root color. Therefore, there is a concern that *P. thunbergii* is sensitive to waterlogging and information on the effect of waterlogging is of urgent need.

Previous research works have found that species with high tolerance show morphological and physiological responses to waterlogging, such as the production of lenticels (Kozlowski and Pallardy, 2002; Shimamura et al., 2010) and adventitious roots (Yamamoto et al., 1995; Li et al., 2006; Steffens and Rasmussen, 2016) which are responses that support the entrance and transportation of oxygen. On the other hand, for species that are sensitive to waterlogging, avoidance responses are not observed and under long-term waterlogging, inhibition of fine root growth (Schmull and Thomas, 2000), induced root decay (Colin-Belgrand et al., 1991), and decrease in root biomass have been reported (Kozlowski, 1997). However, these results are obtained from relatively strong waterlogging stress conditions where the waterlogging depth reaches the soil surface and the whole root system is exposed to waterlogging.

The impact of waterlogging stress on plants is highly dependent on factors such as duration (Pryor et al., 2006), timing (Wang et al., 2015; Roitto et al., 2019), and depth (Jiang and Wang, 2006) of waterlogging. Under natural conditions, waterlogging depths varies. On the other hand, fine roots show high plasticity to environmental factors (Ostonen et al., 2007). Therefore, when the waterlogging is “partial (localized),” it is suggested that fine root growth responses will differ to when the whole root system is exposed to waterlogging. It is hypothesized that under partial waterlogging, localized growth is triggered, where growth is inhibited under waterlogging, and it is continued under non-waterlogged soils. Furthermore, as partial waterlogging would be less stress, responses and adaptions that could not be observed under strong waterlogging conditions would be elucidated, enabling a better understanding of waterlogging response for sensitive species.

Fine roots (diameter < 2 mm) play a vital role in plant growth and survival by absorbing water and nutrients from the soil. Hence, the impact and the damage of waterlogging on fine roots strongly influence these functions, which are essential for above-ground activity. From previous studies, above-ground activity such as transpiration is generally decreased under waterlogging due to the decrease in root water absorption and/or stomatal conductance caused by the decrease in root hydraulic conductance (Aroca et al., 2012). Therefore, to understand the effect of waterlogging on fine roots, not only growth (mass) but also the function (quality) in relation with above-ground activity must be evaluated.

Our research objective is to elucidate effects of different waterlogging depths on fine root growth distribution, fine root morphology, and transpiration of *P. thunbergii* seedlings. Our hypotheses are as follows:

(1) Under partial-waterlogging, fine root growth of *P. thunbergii* seedlings is inhibited at waterlogged bottom soil, whereas fine root can continue to grow at non-waterlogged top soil, likewise to control.
(2) Transpiration of *P. thunbergii* seedlings is decreased under full waterlogging, and the decrease in transpiration is more significant compared to partial waterlogging as the whole root system is exposed to waterlogging.

## Materials and Methods

### Plant Material and Waterlogging Treatment

Two-year-old *P. thunbergii* seedlings were purchased from a commercial tree nursery. At the end of March, 45 seedlings were planted in 1/2,000 a Wagner pots (500 cm^2^, depth 30 cm with a drainage hole) with Akadama soil deriving from the loamy B horizon of an Andisol (Nagakura et al., 2004). After transplantation, seedlings were grown under natural conditions at the experimental nursery of Tohoku Research Center, Forestry and Forest Products Research Institute. After 2 weeks, a 2,000-fold diluted liquid fertilizer (N:P: K = 6, 10, and 5%, Hyponex Japan, Osaka, Japan) was applied with 1 L of water. The amount of nutrients given per pot was approximately 30, 50, and 25 mg of N, P, and K, respectively. Pots were placed on wooded boards and were watered regularly (once a week, 2–4 L) until the waterlogging treatment began. The experimental period was approximately 6 months, from the end of March 2019 until the beginning of October 2019. Since there is a possibility that waterlogging indirectly affects fine root growth through suppressed aboveground growth, waterlogging treatments were started at the beginning of August when shoot and needle elongation had stopped in order to minimize such indirect effects (Table 1). Furthermore, it was aimed at a time when temperatures are high and seedlings are more likely to experience water stress.

**TABLE 1:** Seedlings size after transplantation, before, and after waterlogging treatment.

**Measurement**	**Treatment**	**After transplantation**	**Before WL treatment**	**At end of treatment**
Stem base	Control	7.9 ± 0.3*a*	10.1 ± 0.2*a*	11.4 ± 0.2*a*
diameter (mm)	Partial-WL	8.0 ± 0.2*a*	10.2 ± 0.1*a*	11.9 ± 0.1*ab*
	Full-WL	8.0 ± 0.2*a*	10.1 ± 0.2*a*	12.4 ± 0.2*b*
Height (cm)	Control	23.2 ± 1.0*a*	36.7 ± 1.1*a*	38.3 ± 1.2*a*
	Partial-WL	21.3 ± 0.5*a*	37.0 ± 0.7*a*	38.1 ± 0.7*a*
	Full-WL	22.2 ± 0.5*a*	36.0 ± 1.4*a*	37.2 ± 1.2*a*

*Mean (±SE) of stem base diameter and height of seedlings after transplantation (April 18th), before (August 2nd) and right before the waterlogging treatment ended (September 25th, *n* = 15) for control, partial waterlogging (partial-WL) and full waterlogging (full-WL). Results of the Tukey–Kramer’s multiple comparison test are shown with different italic letters indicating statistical differences between treatments (*p* < 0.05).*

In our experiment, two different waterlogging depths were set. For full waterlogging (full-WL), the drainage hole was blocked and water was added until the water table reached the soil surface (waterlogging depth = 26 cm from the bottom). For partial waterlogging (partial-WL), the waterlogging depth was set at 15 cm from the bottom of the pot. This was done by setting the end of the drainage tube at 15 cm height of the pot so that the water would overflow from the set depth (Supplementary Figure 1). The waterlogging depth was maintained by adding water at 1–2 day interval. For control, the drainage hole was kept open and was watered regularly throughout the experiment period according to the weather (at least once a week, 2–4 L). The waterlogging treatment was done from the beginning of August till the beginning of October (2 months). Each treatment was consisted of 15 seedlings.

### Soil Oxidation–Reduction Potential

The soil oxidation–reduction potential (Eh, mV) was measured by a platinum electrode, a reference electrode, and a logger (FV-702, Fujiwara Scientific Co., Ltd., Tokyo, Japan) (Hara, 2013a,b). Probes were set in pots for the control and full-WL treatment, respectively (*n* = 2). The probes were placed at approximately 10 cm depth from the soil surface. Eh vales were recorded at 2–5 day intervals.

### Root Measurements

In total, three in-growth cores (diameter, 32 mm; height, 30 cm; 2 mm mesh) were used for each pot. All in-growth cores were placed in the pot at the time of transplanting the seedlings. In each pot, two in-growth cores were placed on each side of the seedling, approximately 10 cm apart from the seedling and were used to measure fine root growth during the whole experimental period (IG_total_). The third in-growth core was also placed approximately 10 cm from the seedling, perpendicular to the other two IG_total_. This in-growth core (IG*_wl_*) was covered with a thin plastic sheet to prevent root penetration before the waterlogging treatment. The plastic sheet was carefully taken away at the time of starting the waterlogging treatment, enabling fine roots to penetrate the in-growth core during the waterlogging period.

All in-growth cores were harvested at the beginning of October and stored at 4°C until further analysis. In-growth cores were separated into two sections, the “top part (11 cm from the soil surface)” and “bottom part (15 cm from the bottom of the pot)” (Supplementary Figure 1). For control, both the top and the bottom part were free from waterlogging. For full-WL, both the top and the bottom part were waterlogged. On the other hand, at partial-WL, the top part was free from waterlogging and the bottom part was waterlogged. Fine root growth, morphology, and brightness were analyzed separately for the top and the bottom part.

The soil from the in-growth cores was thoroughly washed out with water on a very fine sieve (sieve aperture, 250 μm). Fine roots were carefully picked from the sieve and carefully washed with a brush. After washing, 10 seedlings from each treatment were randomly chosen and fine roots with a flatbed scanner (GT-X980, EPSON). Scans were made without any image correction and under the same light conditions at 800 dpi. After scanning, fine roots were dried at 70°C for 72 h, and then measured for dry weight (W*_dr_*). For the left five seedlings from each treatment, only W*_dr_* was measured. Fine root growth (mg cm^–3^) per pot was calculated from W*_dr_* in IG_total_ and IG*_wl_*. For IG_total_, fine root growth of each seedling was calculated by adding W*_dr_* of the two in-growth cores and dividing it by the total soil volume of the in-growth core.

The scanned images were analyzed with WinRHIZO Pro (2012b) (Regent Instruments, Inc., Quebec, Canada) for root length (L*_r_*, cm), root projected area (A*_pr_*, mm^2^), and root volume (V*_r_*, cm^3^). Mean root diameter (D*_r_*), specific root length (SRL), and RTD were obtained for evaluating fine root morphology and were calculated from the following equations:

$$D_{r}\,\left(m\,m\right)=\frac{A_{p\,r}}{L_{r}}$$

$$S\,R\,L\,\left(m\,g^{-1}\right)=\frac{L_{r}}{W_{d\,r}}$$

$$R\,T\,D\,\left(g\,c\,m^{-3}\right)=\frac{W_{d\,r}}{V_{r}}$$

For fine root morphology obtained from IG_total_, D*_r_* was calculated by dividing total A*_pr_* by total L*_r_* from the two in-growth cores. For SRL, it was calculated by dividing total L*_r_* by total W*_dr_* of the two in-growth cores. For RTD, it was calculated by dividing total W*_dr_* by total V*_r_* of the two in-growth cores.

For root color, root brightness was evaluated from the scanned images by image analysis carried out by Image J. A brightness histogram (0–255) of the scanned images was calculated as area ratio of the total number of pixels showing root region. Regions showing roots were determined by binarization of images, and brightness was set as the average of colors (R, G, B).

### Transpiration and Needle Traits

Transpiration was estimated by measuring evapotranspiration and evaporation from pots, where the former and latter were estimated by weighing water loss from pots with seedlings (*n* = 15 per treatment) and without seedlings (*n* = 3), respectively. Days of measurement were chosen so that measurement days were all fully sunny and rainless. Before the transpiration measurement, waterlogging was temporally released. Then, all pots were watered until the water ran out from the drainage hole at the bottom to ensure that they were well-watered. Measurements were done during nighttime (20:00–21:30) to minimize transpiration and evaporation during the measurement period. Pots were measured for the first weight and were re-weighed the next night for the second weight. Transpiration per seedling (T, kg pot^–1^) was estimated from the following equation by using the first weight and second weight of pots with seedlings (M*_s1_*, M*_s2_*) and without seedlings (M*_ws1_*, M*_ws2_*);

$$T\,\left(k\,g\,p\,o\,t^{-1}\right)\,=\left\{M_{s\,1}\,\left(k\,g\,p\,o\,t^{-1}\right)-M_{s\,2}\,\left(k\,g\,p\,o\,t^{-1}\right)\right\}-\left\{M_{w\,s\,1}\,\left(k\,g\,p\,o\,t^{-1}\right)-M_{w\,s\,2}\,\left(k\,g\,p\,o\,t^{-1}\right)\right\}$$

Here, “M*_S1_*–M*_S2_*” and “M*_WS1_*–M*_WS2_*” correspond to evapotranspiration and evaporation, respectively. For evaporation, the averaged value obtained from the three pots without seedlings was used. For some pots of full-WL, transpiration after 4 and 8 weeks of waterlogging showed negative values, where the averaged evaporation exceeded evapotranspiration (−0.03 to −0.005 kg). For these pots, transpiration was calculated as zero (six pots). After measurements, pots were filled with water once again for partial-WL and full-WL. In total, pots were released from waterlogging for 1.5 days during the transpiration measurement.

Dry weight of total needles, and fresh and dry needle weight were weighed for current year needles randomly sampled (15–20 needles) from the top part of current year shoot right after seedling harvest in October (*n* = 10–11). Sampled needles were scanned (800 dpi) and then were dried in an oven at 80°C for 72 h before weighing for dry weight. Needle water content (NWC) and leaf mass per area (LMA) were measured to evaluate needle traits. NWC was calculated as follows using W*_fn_* (fresh needle weight, g) and W*_dn_* (dry needle weight, g),

$$NWC\left(\%\right)=\frac{W_{f\,n}-W_{d\,n}}{W_{f\,n}}\times 100$$

Needle area was calculated as needle projected area (A*_pn_*, m^2^), which was obtained from Image J. LMA was calculated as follows,

$$L\,M\,A\,\left(g\,m^{-2}\right)=\frac{W_{d\,n}}{A_{p\,n}}$$

### Statistical Analysis

The Levene’s test was used to check homogeneity of variance. When homogeneity was ensured, statistical difference between treatment groups was tested by Tukey–Kramer’s multiple comparison test, if not Steel–Dwass multiple comparison test was used. All analysis was done using R (version 3.6.1, The R foundation for Statistical Computing Platform). For fine root growth and morphology, the difference between treatment groups was tested separately at the top and bottom part. One-way ANOVA was used to test the difference between control and partial waterlogging for IG*_wl_*.

## Results

### Soil Oxidation–Reduction Potential

**FIGURE 1:**
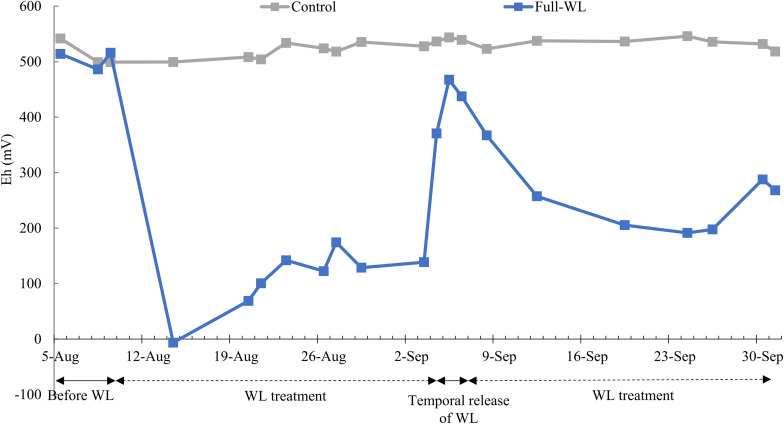
Mean soil oxidation–reduction potential of control and full waterlogging (WL) (*n* = 2).

Soil oxidation–reduction potential (Eh, mV) for full-WL was decreased after approximately 5–7 days after the waterlogging treatment began and was approximately below 300 mV throughout the experiment, except when the Eh value increased due to the temporal release of waterlogging for transpiration measurement. The Eh value decreased again within 1 week, after refilling the pot with water. For control, Eh value was approximately 500–550 mV throughout the experiment (Figure 1).

### Fine Root Growth

**FIGURE 2:**
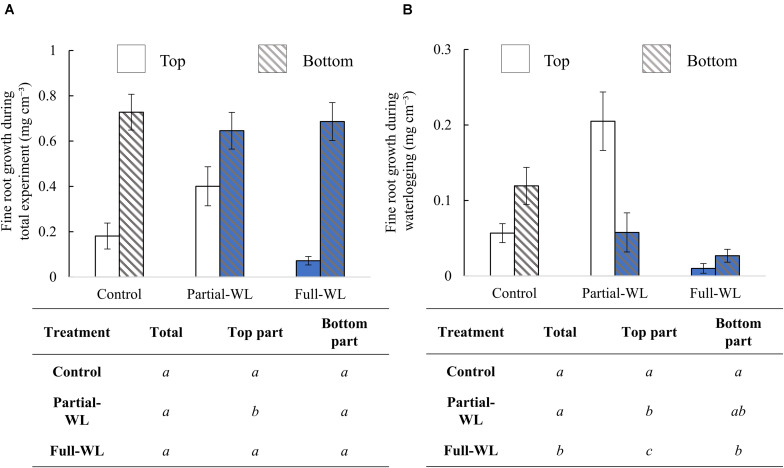
Mean fine root growth (±SE) during **(A)** the whole experiment period (IG_total_) and **(B)** waterlogging treatment period (IG*_wl_*) (*n* = 15 for each treatment and position). Solid bars are results from the top part (11 cm from soil surface), and hatched bars are results from the bottom part (15 cm from the bottom). Bars which are colored with blue are results obtained from waterlogged soils. The result of Tukey–Kramer’s multiple comparison test or Steel–Dwass multiple comparison test (IG_total_: top, IG*_wl_*: total and top part) is shown in the table below the figures. Statistical difference was tested among treatments at the top part, bottom part, and total (top + bottom), respectively. Different italic letters show statistical difference (*p* < 0.05).

Comparing results of IG_total_ between treatment groups, total fine root growth (top + bottom) did not differ between treatment groups. However, at the top part (11 cm from the soil surface), fine root growth was significantly increased at partial-WL (Figure 2A) compared to control. At the bottom part (15 cm from the bottom), fine root growth did not differ among treatments.

From IG*_wl_*, it was found that fine root growth during waterlogging was decreased for full-WL (Figure 2B) at both the top and the bottom part. For partial-WL, total fine root growth did not differ from control. However, compared to control, fine root growth was significantly increased at the top part.

### Fine Root Brightness and Morphology

**FIGURE 3:**
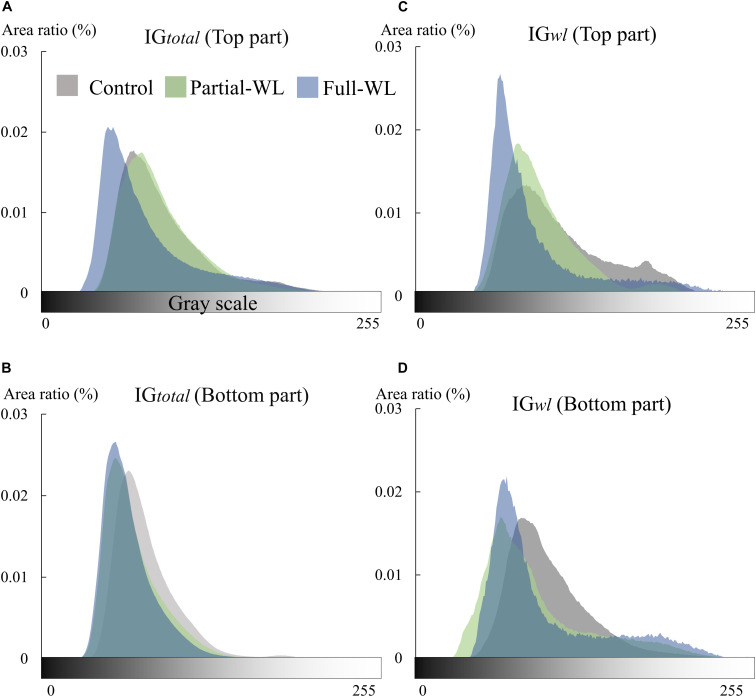
Histogram of root brightness (0–255) of IG_total_ (*n* = 7–10) and IG*_wl_* (*n* = 8–10) at the top (11 cm from the soil surface) and bottom (15 cm from the bottom), respectively **(A–D)**. Gray, green, and blue show results of control, partial waterlogging (partial-WL), and full waterlogging (full-WL), respectively.

Fine roots of *P. thunbergii* were generally brown or relatively dark brown at the top and bottom parts of control. Using image analysis, root color was quantitatively analyzed as root brightness (Figures 3A–D). Concerning IG_total_, control and partial-WL showed a similar histogram peak at the top part where it was not waterlogged for these two treatments. On the other hand, at the bottom part of partial-WL and full-WL, root brightness showed a similar peak, where the histogram peak was at a darker brightness compared to control. For IG*_wl_*, similar results to IG_total_ were observed. At the top part for control and partial-WL, a similar histogram peak was observed. At the bottom, the histogram peak for partial-WL and full-WL was darker compared to control.

Concerning fine root morphology, mean D*_r_*, SRL, and RTD were evaluated from image analysis (Table 2). For IG_total_, D*_r_* was increased compared to control at the bottom part of partial-WL and full-WL, where it was waterlogged. SRL did not differ among treatments at both the top and the bottom part. RTD was decreased compared to control at the bottom part for full-WL.

**TABLE 2:** Fine root morphology obtained from IG_total_.

**Position**	**Treatment**	**Root diameter (mm)**	**SRL (m g^–1^)**	**RTD (g cm^–3^)**
Top part	Control (*n* = 9)	0.45 ± 0.03*a*	27.0 ± 2.0*a*	0.25 ± 0.02*a*
	Partial-WL (*n* = 10)	0.52 ± 0.01*a*	23.7 ± 4.0*a*	0.24 ± 0.03*a*
	Full-WL (*n* = 7)	0.52 ± 0.02*a*	22.5 ± 1.7*a*	0.22 ± 0.01*a*
Bottom part	Control (*n* = 10)	0.42 ± 0.01*a*	26.0 ± 2.0*a*	0.29 ± 0.02*a*
	Partial-WL (*n* = 10)	0.49 ± 0.02*b*	23.3 ± 1.1*a*	0.24 ± 0.01*ab*
	Full-WL (*n* = 9)	0.50 ± 0.02*b*	22.3 ± 2.0*a*	0.24 ± 0.01*b*

*Mean (±SE) root diameter, specific root length (SRL), and root tissue density (RTD) obtained from IG_*total*_ (*n* = 7–10) of control, partial waterlogging (partial-WL), and full waterlogging (full-WL). Values in black show results obtained from non-waterlogged soils, and values in blue show results obtained from waterlogged soils. Statistical difference among treatments was tested at the top part and the bottom part, respectively. Results of the Tukey–Kramer’s multiple comparison test or Steel–Dwass multiple comparison test (top and bottom of RTD) are shown with different italic letters indicating statistical differences between treatments (*p* < 0.05).*

For IG*_wl_*, D*_r_* was increased compared to control at the top part of partial-WL (Table 3). SRL was decreased at the top part for partial-WL. At the bottom part, difference between control and partial-WL was not observed. RTD did not differ between control and partial-WL at both the top and bottom part. For full-WL, fine root morphology could not be evaluated as hardly any fine roots could be obtained from the in-growth cores.

**TABLE 3:** Fine root morphology obtained from IG*_wl_*.

**Position**	**Treatment**	**Root diameter (mm)**	**SRL (m g^–1^)**	**RTD (g cm^–3^)**
Top part	Control (*n* = 8)	0.40 ± 0.02	37.3 ± 3.8	0.23 ± 0.01
	Partial-WL (*n* = 9)	0.51 ± 0.02***	28.1 ± 2.1*	0.23 ± 0.05
Bottom	Control (*n* = 10)	0.44 ± 0.03	36.4 ± 3.9	0.22 ± 0.01
part	Partial-WL (*n* = 9)	0.53 ± 0.05	30.8 ± 4.3	0.19 ± 0.02

Mean (±SE) root diameter, specific root length (SRL), and root tissue density (RTD) obtained from IG*_*wl*_* (*n* = 8–10) of control and partial waterlogging (partial-WL). Values in black show results obtained from non-waterlogged soils, and values in blue show results obtained from waterlogged soils. Statistical difference was tested between treatments at the top part and bottom part, respectively. Results of one-way ANOVA are denoted as: *p < 0.05; ***p < 0.001. Results from full-WL are not shown as hardly any roots could be obtained from in-growth cores.

### Transpiration and Needle Traits

**FIGURE 4:**
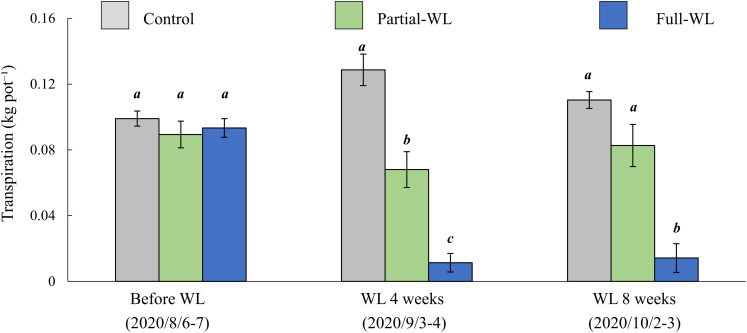
Mean transpiration (±SE) before waterlogging treatment (August 8–6), 4 weeks after waterlogging (September 3–4), and 8 weeks after waterlogging (October 2–3). Gray bars, green bars, and blue bars are the result of control, partial waterlogging (partial-WL), and full waterlogging (full-WL), respectively (*n* = 15). Results of the Tukey–Kramer’s multiple comparison test are shown with different italic letters indicating statistical differences (*p* < 0.05) between treatments at each measurement period.

Transpiration was measured before waterlogging, after 4 and 8 weeks of waterlogging (Figure 4). Before the waterlogging treatment, transpiration did not differ among treatments. After 4 weeks of waterlogging, transpiration was decreased at both partial-WL and full-WL, and full-WL showed a smaller value compared to partial-WL. After 8 weeks of waterlogging, only full-WL was significantly decreased compared to control and partial-WL and there was no significant difference between control and partial-WL.

Results of total needle dry weight and current year needle traits are shown in Table 4. The total dry weight of needles did not differ among treatments. On the other hand, concerning current year needle traits, NWC was significantly decreased at full-WL, and values did not differ between control and partial-WL. LMA was significantly increased at partial-WL and full-WL, and the highest value was observed at full-WL.

**TABLE 4:** Traits of current year needles.

**Treatment**	**Total needle dry weight (g)**	**NWC (%)**	**LMA (g m^–2^)**
Control	30.7 ± 1.3*a*	60.9 ± 0.4*a*	268.2 ± 8.7*a*
Partial-WL	27.5 ± 3.4*a*	59.2 ± 0.5*a*	296.1 ± 6.0*b*
Full-WL	28.5 ± 2.5*a*	53.1 ± 0.5*b*	346.0 ± 8.8*c*

*Mean (±SE) values of total needle dry weight (g), needle water content (NWC), and leaf mass per area (LMA) of the control, partial waterlogging (partial-WL), and full waterlogging (full-WL), respectively (*n* = 10–11). Total needle dry weight was obtained from whole seedlings. Needle water content and leaf mass per area were obtained from randomly sampled current year needles. Results of the Tukey–Kramer’s multiple comparison test are shown with different italic letters indicating statistical differences (*p* < 0.05).*

## Discussion

In this study, soil oxidation–reduction potential (Eh) for full-WL was decreased to <300 mV throughout the period of the waterlogging treatment (Figure 1), with the exception when Eh increased during the temporal release of waterlogging for transpiration measurement. This suggests that full-WL created an oxygen-limited, anaerobic/hypoxic condition compared to control (Pearsall and Mortimer, 1939; Pezeshki and DeLaune, 2012). Increment of stem base diameter at full-WL also indicated that *P. thunbergii* seedlings were affected by waterlogging (Table 1). Previous studies on other pine species such as *Pinus densiflora* reported that stem diameter increment under waterlogging was due to increased bark thickening (stem hypertrophy) (Yamamoto et al., 1987). Therefore, it is suggested that the stem base diameter increase observed at full-WL was a similar response to *P. densiflora*.

Under full-WL, fine root growth was severely inhibited, suggesting that fine roots of *P. thunbergii* are relatively sensitive to waterlogged conditions (Figure 2B). Under partial-WL, fine root growth was suppressed only at the bottom part where it was waterlogged. In contrast, at the top part, fine root growth was significantly increased compared to values of both control and full-WL. These results support hypothesis (1), although the enhancement of fine root growth compared to control at top part was not expected before the experiment. This result indicates that fine root growth of *P. thunbergii* shows high plasticity in their vertical distribution under partial waterlogging.

From field survey, Hirano et al. (2018) reported that *P. thunbergii* growing at shallow groundwater level (sea side) allocates more biomass to the root system, especially to horizontal roots (age 32–58 years). This results in a “plate root system” with shallow vertical roots and long horizontal tap roots. In contrast, at deep groundwater level (land side), deeper vertical roots presented a “tap root system.” For partial-WL, although fine root growth was inhibited under waterlogging at the bottom part, it was enhanced at the top part, resulting in a similar shape to the “plate root system.” Although our results were obtained from a short-term experiment, it reflects the high plasticity of root systems observed in mature trees of *P. thunbergii* at different groundwater depths under natural conditions.

Although high plasticity was observed concerning the spatial distribution of fine root growth under partial waterlogging, response in root morphology mainly indicated root damage. New roots are generally white and turn brown or dark brown with development (Comas et al., 2000; Wells and Eissenstat, 2001). On the other hand, fragile black roots generally indicate dead roots (McClaugherty et al., 1982). Wang et al. (2015) reported that roots turn black under waterlogging for *Betula pendula* and *Betula pubescens* seedlings, and the proportion of black-colored roots was increased under waterlogging possibly due to the deposition of iron. As the deposition of iron/manganese oxides on roots is suggested to interfere with nutrient uptake (Levan and Riha, 1986), the darkening of root color may be indicating damage of fine roots such as the decrease in water absorption function, as transpiration was also decreased under waterlogging. Furthermore, as the darkening in root color and the decrease in RTD was observed at the bottom part of full-WL (Figure 3B and Table 2), not only malfunction but root death was also suggested.

Concerning mean D*_r_*, it was increased at the bottom part for full-WL (Table 2). This increase in D*_r_* was possibly due to the decrease in branching and number of root tips due to the inhibition of growth under waterlogging, or the shedding of root tips due to root death, leaving only the relatively thicker fine roots (Repo et al., 2017).

The increase in D*_r_* was also observed at the top part of partial-WL for IG*_wl_* (Table 3). Here, it is suggested that this increase in D*_r_* is due to a different mechanism than the increase under waterlogging, as the top part of partial-WL is not waterlogged and change in root color was not observed. Under non-waterlogged soils, the increase in D*_r_* may have been due to the production of pioneer roots. Pioneer roots are roots with a larger diameter, suggested to be more readily to defend against abiotic challenges and long exploration in soils (Zadworny and Eissenstat, 2011). Therefore, as the root system was partially under waterlogging stress, pioneer roots may have been preferentially produced to explore soil at the top part and make a new root system to compensate for the damaged roots at the bottom part.

Although total needle dry weight did not differ among treatments, transpiration was significantly decreased at both partial-WL and full-WL after 4 weeks of waterlogging, in which transpiration for full-WL was more decreased than partial-WL (Figure 4). This result followed our hypothesis (2) and other waterlogging research measuring transpiration (Rodríguez-Gamir et al., 2011; Repo et al., 2016). It is suggested that transpiration was decreased due to root damage such as the decrease in water absorption function and/or root death, which was indicated by the decrease in root brightness and the decrease in RTD.

At partial-WL, transpiration recovered to a value which did not differ from control after 8 weeks of waterlogging (Figure 4). It is assumed that this recovery was enabled by the enhanced growth of new fine roots at the top part (Figure 2B), and this worked to recover the balance between water absorption by fine roots and transpiration. Under partial waterlogging, it is suggested that *P. thunbergii* took the strategy of compensation growth of fine roots against waterlogging stress. This result supports research results where it was reported that investments are made to roots where roots can achieve most nutrient and water acquisition (Hodge, 2004; Dresbøll et al., 2013).

Needle traits of current year needles were also affected by waterlogging. NWC of current year needles was significantly decreased at full-WL, possibly due to water stress caused by the decrease in water absorption by fine roots, indicating that needles were experiencing conditions similar to drought stress. LMA was significantly increased at both partial-WL and full-WL (Table 4). Under waterlogging, the reason for the observed increase has been reported to be the accumulation of starch and/or soluble carbohydrates, as carbon fixation exceeds carbon demand for growth (Poot and Lambers, 2003) and root demand is significantly decreased (Gravatt and Kirby, 1998). Although the decrease in fine root growth was significant for full-WL, it was not at partial-WL. Therefore, the increase in LMA may be due to another factor for partial-WL, and further investigations must be made on this point.

In this study, we examined effects using fresh water. We believe that our results give valuable results on effects of different waterlogging depths on *P. thunbergii*. However, at coastal forests, there is not only the concern of waterlogging due to rainfall, but also the additional concern of the rise in sea level due to climate change, resulting in higher groundwater with salinity stress (Plane et al., 2019). Therefore, in future studies, other factors such as waterlogging stress combined with salt stress should be investigated for a better understanding on growth of *P. thunbergii* trees planted at coastal forests.

## Conclusion

This study showed that different depths of waterlogging significantly affect spatial distribution of fine root growth during the waterlogging treatment. Under partial waterlogging, fine root growth was significantly increased at the top part, which enabled the recovery of transpiration. Although *P. thunbergii* is sensitive to waterlogging, according to the depth of waterlogging, it can plastically change fine root growth distribution and compensate for the damaged fine roots by enhancing new fine root growth where it is not waterlogged. This potential acclimation strategy may also explain root system architecture in mature coastal *P. thunbergii* forests under high groundwater level (plate root system), although results of this present study were limited to short-term responses of seedlings.

## Data Availability Statement

The raw data supporting the conclusions of this article will be made available by the authors, without undue reservation.

## Author Contributions

SF, KN, and TT conceived and designed the experiments and wrote the manuscript. SF and KN performed the experiments and analyzed the data. TT supervised the research. All authors read and agreed to the published version of the manuscript.

## Conflict of Interest

The authors declare that the research was conducted in the absence of any commercial or financial relationships that could be construed as a potential conflict of interest.
