# Supplementary figures and images for: Different Waterlogging Depths Affect Spatial Distribution of Fine Root Growth for Pinus thunbergii Seedlings
Source: Front Plant Sci. 2021 Mar 10;12:614764. doi: 10.3389/fpls.2021.614764 (PMC7988193; doi:10.3389/fpls.2021.614764)

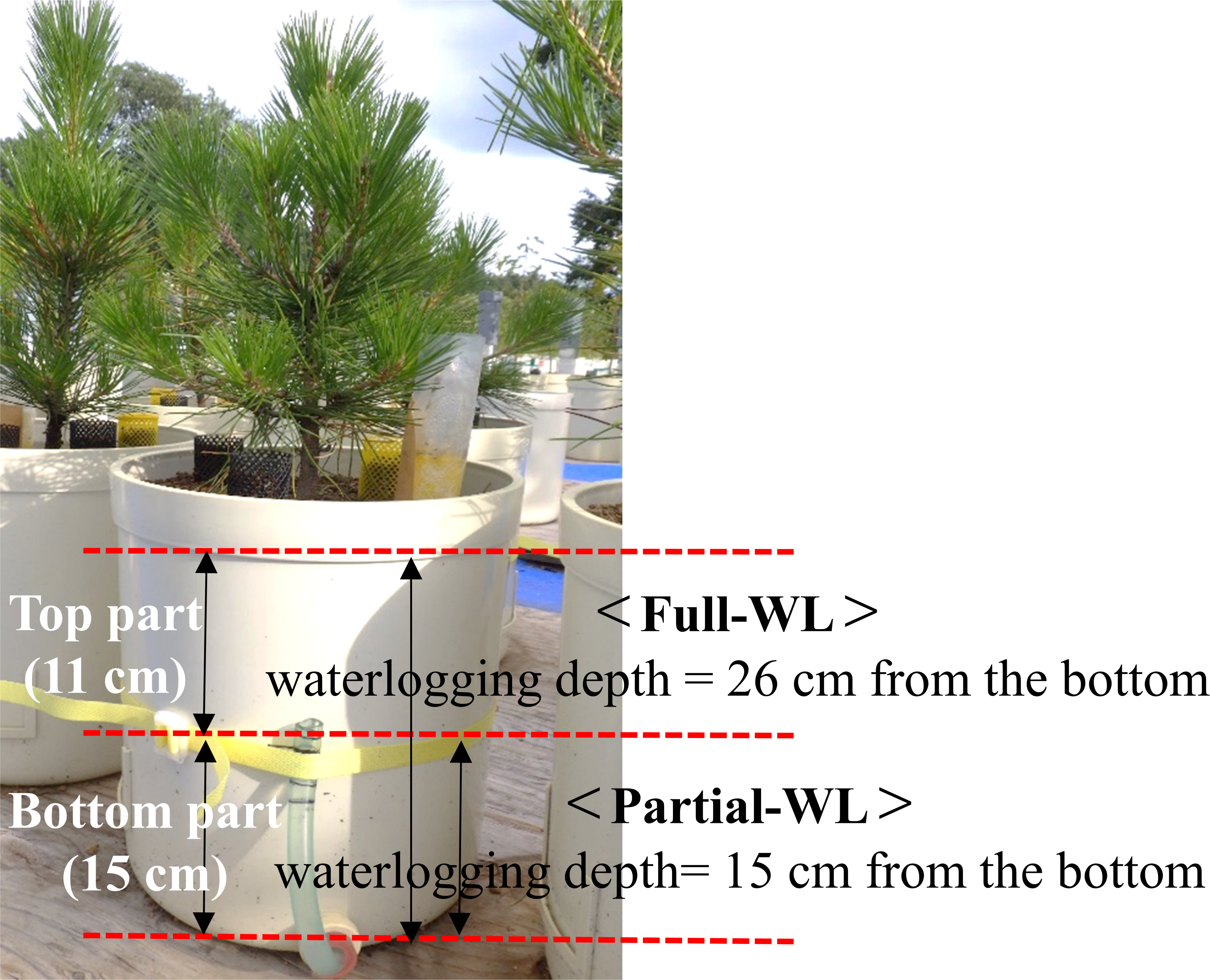

Supplement: Supplementary file 1 [file Image_1.TIF]
